# Treatment of the Posterolateral Tibial Plateau Fractures using the Anterior Surgical Approach

**Published:** 2010-12

**Authors:** Chih-Hsin Hsieh

**Affiliations:** 1 *Department of Orthopedics, Kaohsiung Medical University Hospital, Kaohsiung Medical University, Kaohsiung, Taiwan (ROC);*; 2 *Department of Orthopedics, Ping Tung Hospital, Department of Health, Executive Yuan, Taiwan (ROC)*

**Keywords:** anterior approach, posterior aspect, tibial plateau fracture

## Abstract

Background: Fracture of the posterolateral tibial plateau is relatively uncommon. While surgical treatment by the posterior approach is theoretically ideal, this approach is associated with numerous complications. We describe a series of fractures of the posterolateral tibial plateau treated by the anterior surgical approach. Methods: Fifteen patients with posterolateral tibial plateau fractures were included in this study. All patients were treated operatively using the anterior approach. The quality of fracture reduction was evaluated and functional results were estimated by the Hospital for Special Surgery knee scoring system. Results: The most common cause of fracture was a motor scooter accident (86%, 13 of 15 patients), which may have resulted in the protective front plate of the scooter hitting the knee in the flexion position, causing an axial compression and valgus force, resulting in the fracture of the posterolateral tibial plateau. The average knee motion after surgery was 0–124° of flexion and 14 out of 15 patients (93%) experienced satisfactory articular reduction. There were no postoperative neural or vascular injuries and no wound complications. The average Hospital for Special Surgery knee score was 92 (range, 74–98). Conclusions: In our series, with careful preoperative planning, the anterior approach for the surgical treatment of posterolateral tibial plateau fractures had no complications and was associated with satisfactory outcomes.

## INTRODUCTION

A tibial plateau fracture involving posterolateral fragments is an uncommon injury that is rarely reported in the literature (1–3). Some authors have proposed a posterior surgical approach for this fracture (1, 2). This approach is ideal for fractures of the posterior aspect of the tibial plateau, as it allows for direct open reduction and buttress plate fixation. However, the merits of this approach are superseded by procedural complications. Tao *et al*. (2) described a modified posterolateral approach, which resulted in 5 of 11 patients experiencing a 5° flexion contracture. Chang *et al*. (1) reported the treatment of posterior coronal fractures of the lateral tibial plateau in 8 patients with buttress plate fixation by the direct posterolateral approach. Four patients experienced a flexion lag of 10–20° and 1 patient experienced postoperative peroneal nerve distribution paresthesia. Thus, the ideal treatment for this condition remains unclear. The purpose of this study was to present a case series of patients with posterolateral tibial plateau fractures that were treated by the anterior surgical approach and to evaluate the functional results and complications of the procedure.

## MATERIALS AND METHODS

**Figure 1:**
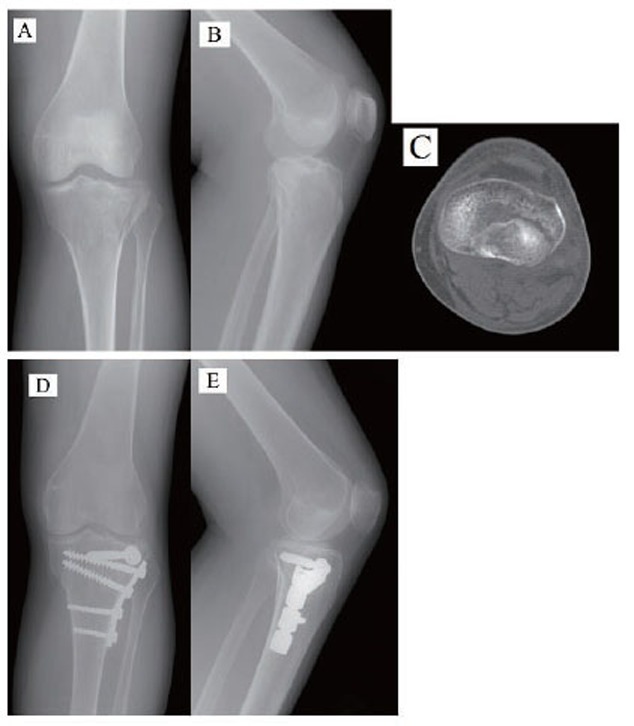
(A–C), Anteroposterior, lateral radiographs, and computed tomography scans of the right knee in a 43-year-old female showing the split-depression fracture of the posterolateral tibial plateau. (D, E), Radiographs showing the fracture reduction and implant position following surgery.

From January 2004 to December 2008, 230 patients with tibial plateau fractures were operated on at our institution. Among these patients, 15 (6%) had fractures of the posterolateral tibial plateau, all of which were followed up for this study. It was a retrospective review. The institutional review board of the “Human Experimental and Ethics Committee” of our hospital approved the study design and written informed consent was obtained from all patients or their relatives. The study included 11 female and 4 male patients, ranging in age from 21–73 years. Patient demographics are shown in Table 1. The average age at the time of injury was 48 years. The fractures involved 6 left knees and 9 right knees. Patient demographics are shown in Table 1. Anteroposterior and lateral radiographic views and computed tomography (CT) scans were assessed before surgery (Fig. 1) and all patients were operated on using the anterior approach.

**Table 1:** Patient demographics

Case No.	Gender	Age (years)	AO/OTA classification	Time from injury to surgery (days)	Cause of injury	Follow-up (months)	Bony union (weeks)	Range of motion (°)	HSS Knee score	Radiological results		
										Articular step-off (mm)	Coronal alignment (°)	Sagittal alignment (°)

1	M	53	41−B1.1 (4)	1	Motor scooter accident	26	12	0–130	94	1	87°	6°
2	F	46	41−B3.1 (2)	1	Motor scooter accident	26	12	0–135	95	0	87°	8°
3	F	43	41−B3.1 (2)	5	Fall from height	25	12	0–125	91	0	90°	7°
4	F	44	41−B3.1 (2)	1	Motor scooter accident	25	8	0–125	93	2	90°	8°
5	M	73	41−B3.1 (2)	1	Automobile accident	24	12	0–120	88	0	89°	9°
6	M	21	41−B1.1 (4)	1	Motor scooter accident	24	12	0–130	93	0	92°	5°
7	F	63	41−B3.1 (2)	4	Motor scooter accident	19	12	0–125	90	1	88°	9°
8	F	56	41−B2.2 (4)	1	Motor scooter accident	18	12	0–130	98	0	86°	6°
9	M	60	41−B2.2 (4)	3	Motor scooter accident	12	12	0–125	95	0	89°	4°
10	F	34	41−B3.1 (2)	1	Motor scooter accident	12	12	0–120	90	0	87°	12°
11	F	53	41−B1.1 (4)	1	Motor scooter accident	12	12	0–120	95	0	89°	10°
12	F	58	41−B2.2 (4)	10	Motor scooter accident	9	12	0–125	90	4	89°	9°
13	F	35	41−B2.2 (4)	1	Motor scooter accident	6	8	0–120	95	0	88°	9°
14	F	48	41−B2.2 (4)	2	Motor scooter accident	6	12	0–125	97	0	89°	14°
15	F	40	41−B2.2 (4)	1	Motor scooter accident	2	8	0–115	74	0	87°	8°

Under general anesthesia, the patient was placed in the supine position with a pneumatic tourniquet around the thigh. Antibiotic prophylaxis with a cephalosporin antibiotic was administered routinely. We made an anterolateral incision starting above the superior pole of the patella and extending distally below the inferior margin of the fracture site. The fascia was incised parallel to the anterior border of the iliotibial tract. We gained intra-articular entry by incising the coronary or infra-meniscotibial ligament and retracted the lateral meniscus superiorly. The origin of the extensor muscles was stripped from the anterolateral aspect of the condyle through an inverted L-shaped incision. We extended the horizontal limb of the incision laterally from the tibial tuberosity and passed the vertical limb of the incision distally just lateral to the crest of the tibia. Thereafter, a cortical window below the area of depression was made. We inserted a periosteal elevator well beneath the depressed articular fragments and the articular fragments were elevated with careful and gentle upward pressure. Using fluoroscopic guidance, the articular surfaces were reduced with the knee in full extension or slight hyperextension. Thereafter, cancellous bone graft, which was obtained from the iliac crest of the patient or the bone bank, was packed into the defect. Multiple Kirschner wires were inserted into the posterolateral popliteal fossa as a temporary fixation for the fragments. Cancellous screws and a contoured buttress plate were applied for definitive fixation. The meniscus was carefully sutured back to its attachment or to the proximal screw. The fascia and the skin were closed over suction drains. A posterior plaster splint was applied post-operatively.

After surgery, a passive motion machine was used for several hours per day and physical therapy with emphasis on muscle strengthening exercises was prescribed. No weight bearing was permitted for 12 weeks. Regular radiographic views were obtained postoperatively, every 4 weeks until the fracture healed and once a year thereafter. The fracture union was defined on the basis of a combination of clinical and radiographic criteria. Clinical criteria were absence of pain or tenderness at the fracture site during weight-bearing. Radiographic criteria was bridging of the fracture site on the anteroposterior and lateral radiographic views. The quality of fracture reduction was evaluated on the basis of three radiographic parameters: articular reduction, coronal alignment, and sagittal alignment. The way that the articular step-off was measured took into account the degree of magnification on x-ray. Postoperative radiographs were reviewed by one independent staff orthopedic surgeon. Fracture reduction was defined as satisfactory if there was an articular step-off of ≤2 mm, the medial proximal tibial angle was 87° ± 5°, and the posterior proximal tibial angle was 9° ± 5° (4). At the final follow-up visit, measurements of the knee range of motion were done and all patients were evaluated using the Hospital for Special Surgery (HSS) knee scoring system (5).

## RESULTS

Thirteen patients were injured in a motor scooter accident (86%), one was injured in an automobile accident, and one was injured in a fall from a height. The mean time from injury to surgery was 2 days (range, 1–10 days) and the average duration of follow-up was 16 months (range, 2–26 months). Bony union occurred at a mean of 11 weeks (range, 8–12 weeks) after surgery. The stability of the knee joint was assessed after osseous anatomy was restored; no ligamentous rupture was observed. All fractures were located on the posterolateral tibial plateau. According to the AO/OTA classification system (6), 6 patients had split-depression fractures that were classified as 41-B3.1 (2). Six patients had pure depression fractures that were classified as 41−B2.2 (4). Additionally, 3 patients had pure split fractures that they were classified as 41−B1.1 (4).

At the final follow-up, the average knee motion was 0–124° of flexion. Fourteen patients had a satisfactory articular reduction (≤2 mm step or gap), but in 1 patient the reduction was graded as imperfect (>2 mm step-off; 6.6%). Among the 14 patients with satisfactory articular reduction, articular reduction was perfect (absolutely no step-off) in 11 patients. The mean medial proximal tibial angle was 88° and the mean posterior proximal tibial angle was 8°. All patients had satisfactory sagittal alignment and coronal alignment. There were no cases of nonunion, malunion, loss of reduction, or wound complications. No patients sustained neural or vascular injuries. In none of the 15 patients, joint space narrowing of the knee, indicative of posttraumatic arthritis, was seen at the final follow-up. The average HSS score (5) was 92 (range, 74–98).

## DISCUSSION

In this study, we have demonstrated the efficacy of the anterior approach in the operative treatment of fractures of the posterior aspect of the lateral tibial plateau. Fractures of the posterior aspect of the lateral tibial plateau are unusual injuries and are seen as a split fracture and/or depression in the posterolateral aspect of the tibial plateau. Fractures in this area are difficult to detect on anteroposterior and lateral radiographs (3). CT is useful for surgical planning and to determine the extent of the posterolateral tibial plateau fracture.

Fracture of the posterolateral tibial plateau has been previously underestimated and its specific fracture pattern is not well described by the Schatzker classification system (7). Mason (8) classified tibial plateau fractures into 6 groups, including undisplaced, central depression, split depression, total depression, split, and comminuted upper end of the tibia fractures. Kennedy *et al*. (9) classified fractures of the lateral tibial plateau into 4 groups as follows: abduction fractures, compression fractures, mixed fractures, and explosive fractures. Compression fractures were further classified as central, anterior, lateral, and posterior. Moore (10) described a radiographic classification of fracture-dislocations of the knee, including split, entire condyle, rim avulsion, rim compression, and four-part fractures.

The AO/OTA classification system (6, 11) classifies posterolateral tibial plateau fractures as being partial articular (41−B). Adding a number in parenthesis depicts the posterior articular surface in a more comprehensive way; 41−B1.1 (4) illustrates a partial articular split fracture of the proximal tibial lateral surface on the posterior aspect on the frontal plane; 41−B2.2 (4) illustrates a proximal tibial partial articular depression fracture of the lateral plateau on the posterior aspect; 41−B3.1 (2) illustrates a proximal tibial partial articular split-depression fracture of the lateral plateau on the postero-lateral part; and 41−B3.1 (4) illustrates a proximal tibial partial articular split-depression fracture of the lateral plateau on the postero-medial aspect. Presently, the AO/OTA system (6, 11) is the most exhaustive and practical classification system for these specific fracture patterns.

In the present study, the most common reason for fractures of the posterior aspect of the lateral tibial plateau was a motor scooter accident. The motor scooter is a light motorcycle with a protective front plate and support for the rider’s feet that is a very popular personal transportation vehicle in our city. When riding the scooter, the driver sits with his/her knee at a >90° angle. When a motor scooter accident occurs, the protective front plate of the scooter may hit the knee in the flexion position, resulting in axial compression and a valgus force being applied to the posterolateral aspect of the tibial plateau, which may result in a fracture of the posterolateral tibial plateau. Associated ligament and soft tissue injuries are rare. However, when a greater force is applied, posterior subluxation of the femur on the tibia may result in the rupture of an anterior cruciate ligament (3).

There are few reports discussing the treatment of posterolateral tibial plateau fractures in the literature (1–3). Non-operative treatment of these fractures is limited, as instability of the knee in flexion is frequently evident, although stability in full extension is common. Daily activities like ascending and descending stairs, rising from a seated position, or many athletic activities require a stable knee in flexion (3). Some studies have described the posterior surgical approach for the treatment of the posterior aspect of tibial plateau fractures (1, 2, 12–15). The posterior approach provides direct exposure of the fracture, enabling fracture reduction under visualization and buttress plate fixation. However, this approach is associated with a high complication rate. The common peroneal nerve at the posterior aspect of the biceps femoris muscle, the popliteal vessels, the saphenous nerve at the posterior aspect of the medial plateau, the medial sural cutaneous nerve, and the tibial nerve in the popliteal fossa may be damaged during direct exposure of the fractures using the posterior approach.

Georgiadis (12) reported the use of a posterior plate to fix the posteromedial fragments in 4 patients. Two patients experienced transient paresthesia of the saphenous nerve and 2 patients experienced a 5° extension lag. De Boeck *et al*. (13) described 7 patients with posteromedial tibial plateau fractures treated via a single posterior approach. The average extension lag was 7° and the average flexion lag was 18°; 1 patient developed deep vein thrombosis. Carlson (15) reported a direct posterior approach through dual incisions in 5 patients with posterior bicondylar tibial plateau fractures. One patient developed deep vein thrombosis and a superficial wound dehiscence, while 3 patients experienced transient saphenous nerve sensory deficits. Thus, for the treatment of posterolateral tibial plateau fractures with the posterolateral approach, a high risk of flexion contracture and peroneal nerve paresthesia has been observed (1, 2).

In our study, using the anterior approach, there were no neural or vascular injuries and no patients sustained flexion contractures of the knee. Furthermore, the articular reduction we observed was imperfect in just one patient and satisfactory in 14 patients. Among those 14 patients, the articular reduction was perfect in 11 patients. The imperfect reduction rate in our study was 6.6%, which is comparable to and slightly better than that reported in other studies (11–12.5%) (1, 2). Although it was difficult to achieve direct reduction for the posterior fragments, good reduction could be accomplished by way of extension or hyperextension of the knee with the aid of fluoroscopy. Bone grafting was required during elevation and reduction of the articular surface for this particular fracture pattern. We used packing cancellous bone graft for the filling of the defect and achieved excellent articular surface restoration and bony union.

In this study we have demonstrated that (a) the anterior approach to the surgical treatment of fractures of the posterior aspect of the lateral tibial plateau can prevent the neurovascular injuries and flexion contractures evident following the posterior approach; (b) the most common cause of this injury was axial compression and valgus force application with the knee in a flexion position during a motor scooter accident; and (c) during reduction of the posterolateral tibial plateau fractures, the knee should be kept in extension or hyperextension to reduce the posterior fragments, and that the appropriate fixative must be applied.
